# Identification of prognosis-related molecular subgroups based on DNA methylation in pancreatic cancer

**DOI:** 10.1186/s13148-021-01090-w

**Published:** 2021-05-12

**Authors:** Xiaoli Yin, Lingming Kong, Peng Liu

**Affiliations:** 1 Department of Radiology, Shengjing Hospital of China Medical University, Shenyang, 110004 China; 2 Department of General Surgery, Shengjing Hospital of China Medical University, Shenyang, 110004 China

**Keywords:** Pancreatic cancer, DNA methylation, Prognosis model, Molecular characteristics

## Abstract

**Background:**

Pancreatic cancer (PC) is one of the most lethal and aggressive cancer malignancies. The lethality of PC is associated with delayed diagnosis, presence of distant metastasis, and its easy relapse. It is known that clinical treatment decisions are still mainly based on the clinical stage and pathological grade, which are insufficient to determine an appropriate treatment. Considering the significant heterogeneity of PC biological characteristics, the current clinical classificatory pattern relying solely on classical clinicopathological features identification needs to be urgently improved. In this study, we conducted in-depth analyses to establish prognosis-related molecular subgroups based on DNA methylation signature.

**Results:**

DNA methylation, RNA sequencing, somatic mutation, copy number variation, and clinicopathological data of PC patients were obtained from The Cancer Genome Atlas (TCGA) dataset. A total of 178 PC samples were used to develop distinct molecular subgroups based on the 4227 prognosis-related CpG sites. By using consensus clustering analysis, four prognosis-related molecular subgroups were identified based on DNA methylation. The molecular characteristics and clinical features analyses based on the subgroups offered novel insights into the development of PC. Furthermore, we built a risk score model based on the expression data of five CpG sites to predict the prognosis of PC patients by using Lasso regression. Finally, the risk score model and other independent prognostic clinicopathological information were integrative utilised to construct a nomogram model.

**Conclusion:**

Novel prognosis-related molecular subgroups based on the DNA methylation signature were established. The specific five CpG sites model for PC prognostic prediction and the derived nomogram model are effective and intuitive tools. Moreover, the construction of molecular subgroups based on the DNA methylation data is an innovative complement to the traditional classification of PC and may contribute to precision medicine development, therapeutic efficacy prediction, and clinical decision guidance.

**Supplementary Information:**

The online version contains supplementary material available at 10.1186/s13148-021-01090-w.

## Background

Pancreatic cancer (PC) is described as the worst malignant solid tumour owing to its rapid progression, high invasiveness, and poor prognosis. Although tremendous advances in several aspects of PC treatment have been recently made, its morbidity and mortality rates still do not show a noticeable decrease, and its 5-year relative survival rate is lower than that of other solid tumour malignancies [1, 2]. PC lethality is determined by its delayed diagnosis, distant metastasis, and easy relapse characteristics; thus, the available curative therapies will be limited to some extent. Nowadays, surgical section is PC potentially curative treatment for patients in the early stage [3]. Therefore, it is necessary to develop new approaches for the prevention and early detection of PC.

The oncological diagnosis has been expanded to include molecular features of cancers, which could serve as an important complement to the common tumour data currently used, such as clinical and pathological information. Identification of specific molecular features in different tumours may contribute to a better elucidation of the underlying aetiology, clinical characteristics, and outcomes of cancers [4, 5]. Previous studies have attempted to explore PC molecular subtype classification to make optimal clinical decisions and therapeutic strategies before the treatment [6]. Collisson et al. proposed the classifications of pancreatic ductal adenocarcinoma in three subtypes (classical, quasi-mesenchymal, and exocrine-like) based on the transcriptional profiles of PC samples; these subtypes showed significant differences in crucial aspects such as clinical survival and therapeutic reaction (7). Moreover, Puleo et al. identified five distinct molecular subtypes of pancreatic ductal adenocarcinoma using the consensus clustering method of gene expression data from 309 paraffin-embedded tissue samples [8], and Follia et al. determined four metabolic subtypes by integrated analysis of glycolysis-related genes. In the latter study, different prognosis and genomic mutations were identified among the four molecular subgroups, which might contribute to the setting of personalised treatments [9]. In addition, Namkung et al. proposed three molecular subtypes that presented significant difference in the prognosis of PC and were based on the microRNA expression profiles of 104 tissue samples [10]. The increase of novel classification methods based on the strength of different omics could contribute to elucidate the underlying mechanisms of oncogenesis and to recognise molecular subtype associated with potential therapeutic targets, enabling the construction of clinically applicable molecular subgroups to complement the current clinical and histopathological criteria.

Growing evidences have demonstrated that the abnormal expression patterns of tumour suppressor or cancer-promoting genes frequently occur in PC tissues, leading to PC tumourigenesis [11, 12]. DNA methylation, which is controlled by an array of DNA methylation transferases and demethylation enzymes, plays a vital role in the epigenetic modifications of cancer [13]. Recent studies have found that DNA methylation may impact on the expression of tumour suppressor genes in early stages of the complex process of tumourigenesis [14]. Meanwhile, the hypomethylation status of various cancer-promoting genes, such as ANK1, MET, ITGA2, and P-cadherin, correlates with high gene expression levels, which will conduce to the occurrence and progression of PC [15–17]. In addition, DNA methylation signatures can be utilised as biomarkers of resistance or sensitivity to a particular drug [18]. The high-frequency rate of epigenetic modifications in tumour results in the generation of diverse gene expression patterns, which can rapidly evolve through drug selection during treatment, leading to the development of drug resistance [19]. Since DNA methylation could play a crucial role in multiple aspects of cancer, several prognosis-related models have been proposed for central nervous system, non-small cell lung, colon, and metastatic prostate cancers [20–23]. Owing to the establishment of specific molecular-based cancer subtypes, the patients could receive a personalised treatment, and benefit from precision medicine. Therefore, it is pivotal to redefine the molecular subtypes of PC based on DNA methylation features, as little has been reported in this topic.

In this study, we conducted in-depth analyses to establish prognosis-related molecular subgroups based on DNA methylation signature. To this end, DNA methylation, RNA sequencing, somatic mutation, copy number variation, and clinicopathological data of PC patients were obtained from The Cancer Genome Atlas (TCGA) dataset [24, 25]. Then, consensus clustering analysis was employed to identify specific prognosis-related molecular subgroups based on the DNA methylation signature. Novel molecular characteristics and mechanisms behind the redefined subtypes were identified. Based on five CpG sites, we constructed a prognostic prediction model and a nomogram model. This new approach to define the molecular subgroups of PC based on DNA methylation profiles might conduct to the recognition of patients heterogeneity and to contribute with guide therapeutic options and clinical decisions to improve the outcomes of PC.

## Results

### Identification of four molecular subgroups based on DNA methylation data

**Fig. 1:**
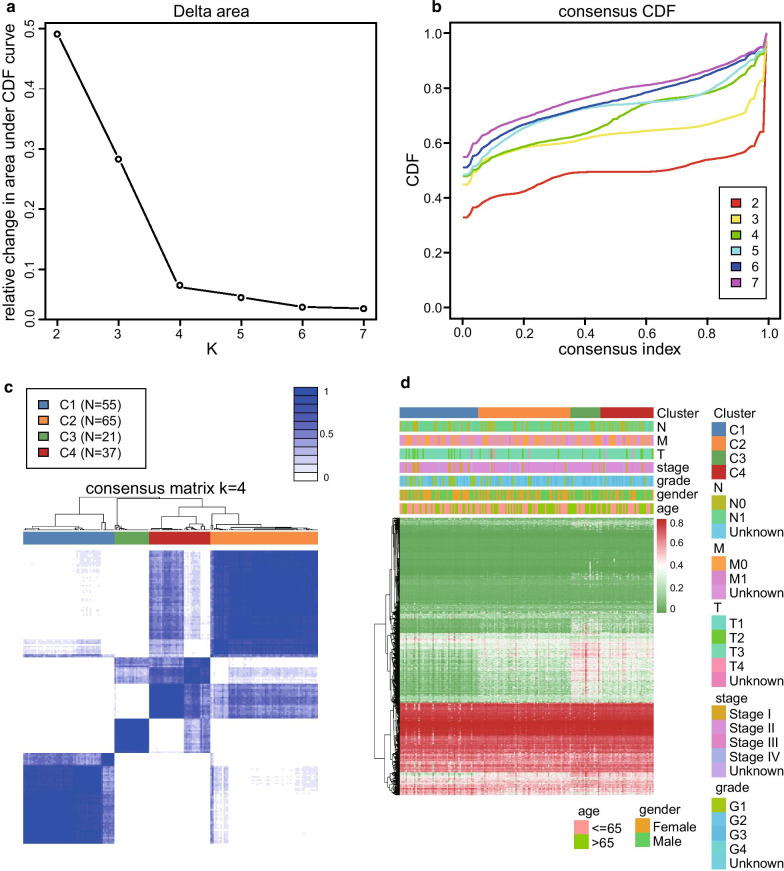
Identification of the four molecular subgroups by using the consensus clustering analysis. **a** Relationship between the relative changes in the area under the cumulative distribution function (CDF) curve and the numbers of the molecular subgroups (K value). **b** Consensus CDF of the different numbers of clusters. The X-axis represents the consensus index. **c** Heat map of the consensus matrix when the total samples are clustered into four molecular subgroups (K = 4). **d** Heat map of 4227 CpG sites data and corresponding clinicopathological information of the four molecular subgroups

The DNA methylation data of 195 PC samples were pre-processed according to the above-described methods. A total of 206,635 CpG sites were selected for the analysis. The univariate Cox proportional hazards regression survival analysis identified 29,879 prognostic CpG sites (*P* < 0.05, Additional file 1: Table S2). Moreover, using a multivariate Cox regression model, we identified 4227 independent prognostic CpG sites for further consensus clustering study (*P* < 0.05, Additional file 1 Table S3). The PC samples with survival period lower than 30 days were excluded from analysis. Finally, a total of 178 PC samples were used to identify distinct molecular subgroups based on the above 4227 CpG sites. According to the criteria of the consensus clustering analysis, the K value was selected when the area under the CDF plot became stabilised without an obvious increment (Fig. 1a, b). Therefore, all the PC samples were clustered into four molecular subgroups. The consensus matrix of the total samples showed that the four subgroups were arranged as four well-defined areas with internal distinctiveness (Fig. 1c). The number of samples in C1, C2, C3, and C4 subgroups was 55, 65, 21, and 37, respectively. The heat map of the 4227 CpG sites data and corresponding clinicopathological information of the total PC samples are shown in Fig. 1d.

### Survival analysis and clinical feature comparison among the four subgroups

**Fig. 2:**
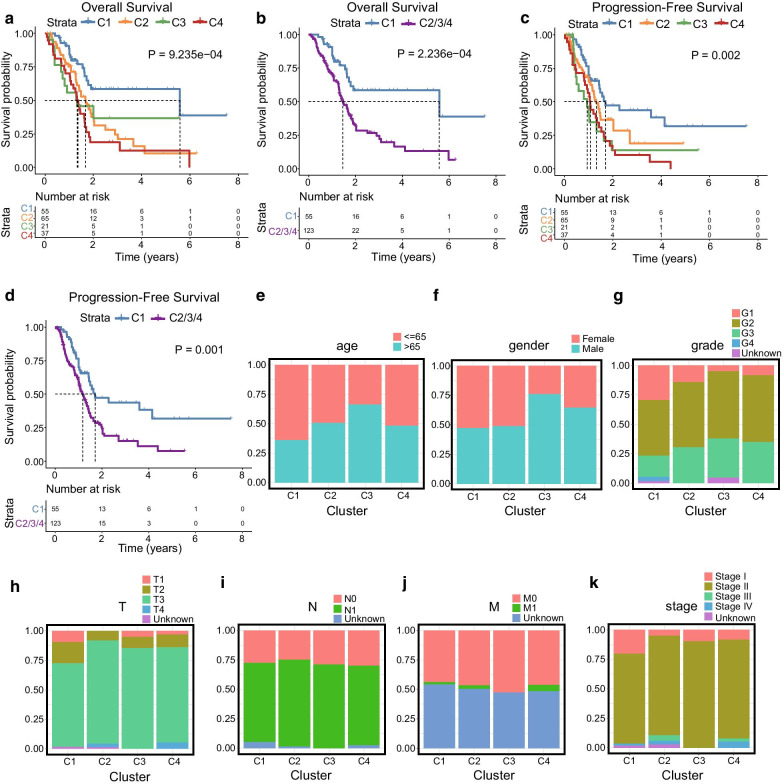
Survival and clinical feature comparisons between the C1, C2, C3, and C4 subgroups. **a** Overall survival analysis among the four subgroups. **b** Overall survival analysis between C1 and C2/3/4 subgroups. **c** Progression-free survival analysis among the four subgroups. **d** Progression-free survival analysis between C1 and C2/3/4 subgroups. **e**–**k** Proportional distribution plots of common clinicopathological information including age (**e**), gender (**f**), grade (**g**), T stage (**h**), N stage (**i**), M stage (**j**), and clinical stage (**k**)

According to the above clustering analysis, the PC samples were distributed in four subgroups (C1, 55 samples; C2, 65 samples; C3, 21 samples; C4, 37 samples). The overall survival analysis showed that there were significant differences among the four subgroups (*P* = 9.235e − 04, Fig. 2a). C1 showed better prognosis than the total samples (C1 vs C2/3/4, *P* = 2.236e − 04, Fig. 2b). Moreover, the progression-free survival analysis indicated that there were significant differences among the four subgroups (*P* = 0.002, Fig. 2c). Similarly, C1 group exhibited the best prognosis and had significant difference compared with the prognosis of the rest of the samples (*P* = 0.001, Fig. 2d). To compare the clinical features of the four subgroups, proportional distribution plots of different clinical features (age, gender, tumour grade, T stage, N stage, M stage, and clinical stage) were generated (Fig. 2e–k). The samples of C1 group tended to be from younger patients (age ≤ 65) with lower tumour grade (G1–G2), and lower T stage (T1–T2). The C3 group was composed by older patients (age > 65 years) and presented an advanced tumour grade (G3–G4). The C2 group showed advanced T stage (T3–T4). These results showed that the clinicopathological features are closely related to the clustering of the subgroups.

### Comparisons of the molecular characteristics among the four subgroups

**Fig. 3:**
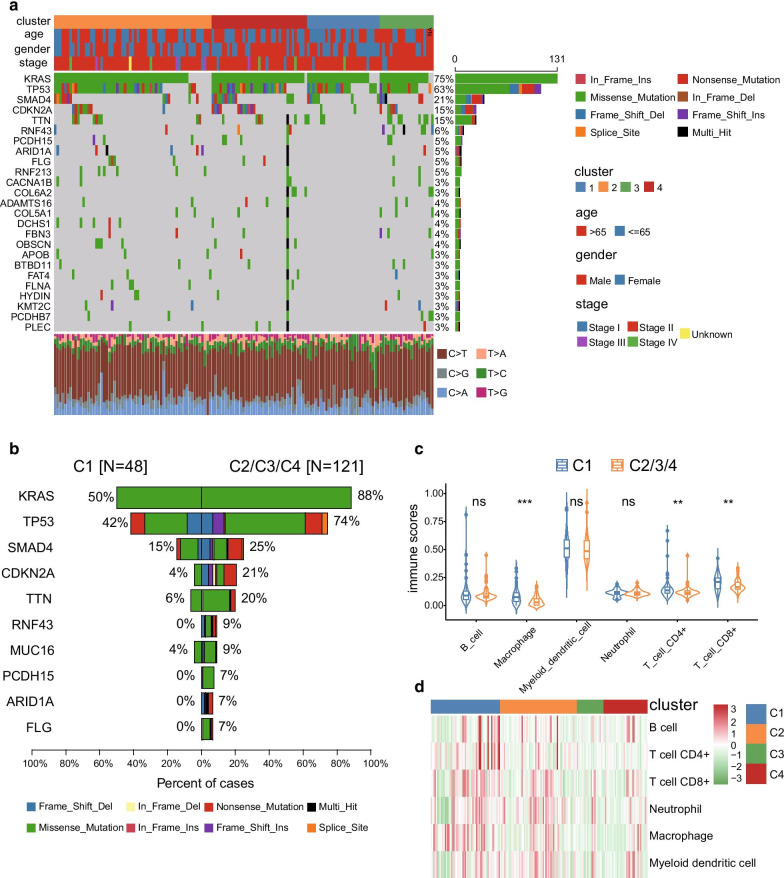
Molecular characteristics analysis between the four subgroups. **a** Oncoplot including the top 25 mutated genes and its mutational frequency of the total samples. **b** Top 10 genes with significant differences in the mutational frequency between C1 and C2/3/4 subgroups. **c** Violin plot of the immune scores between C1 and C2/3/4 subgroups. **d** Heat map of the six types of immune cells of the four molecular subgroups

**Fig. 4:**
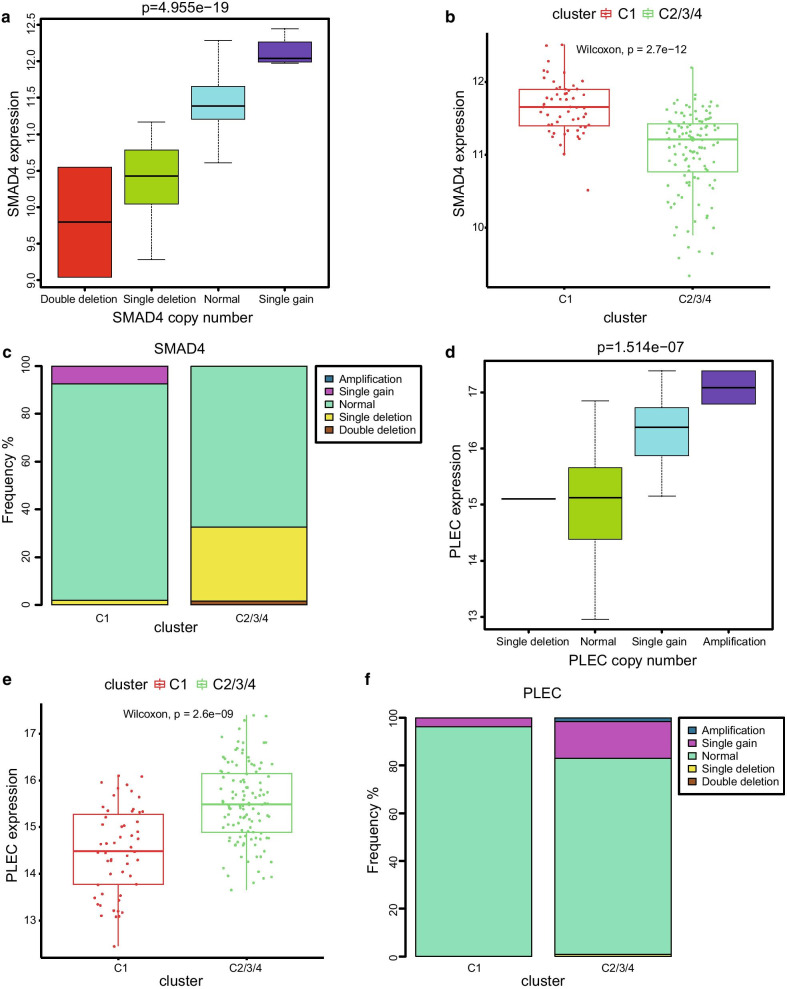
Copy number variation of SMAD4 and PLEC genes between C1 and C2/3/4 subgroups. **a** Correlation analysis between SMAD4 expression level and SMAD4 copy number. **b** SMAD4 expression level between C1 and C2/3/4 subgroups. **c** Frequency of copy number variation of SMAD4 gene in C1 and C2/3/4 subgroups. **d** Correlation analysis between PLEC expression level and PLEC copy number. **e** PLEC expression level between C1 and C2/3/4 subgroups. **f** Frequency of copy number variation of PLEC gene in the C1 and C2/3/4 subgroups

To further investigate the underlying molecular mechanisms behind the prognosis-related subgroups division, we performed a mutational spectrum analysis of the four subgroups. An oncoplot containing the top 25 mutated genes and their mutational frequency regarding the total samples is shown in Fig. 3a. KRAS, TP53, SMAD4, and CDKN2A were classical cancer-related genes that showed close relationships with the tumour initiation and progression process. The mutational frequency of these genes in the C1 subgroup was significantly lower than that of the C2/3/4 subgroups (Fig. 3b, Additional file 1: Table S4). Further structural variation analyses of these mutated genes were performed based on the copy number variation data. The SMAD4 gene is a known tumour suppressor gene for PC. Interestingly, our results indicated that SMAD4 expression was significantly correlated with its copy numbers (Fig. 4a). SMAD4 expression was higher in the C1 subgroup than in the C2/3/4 subgroups, and the frequencies of single and double deletion were lower in the C1 subgroup (Fig. 4b, c). *PLEC* could serve as an ideal biomarker for early detection of PC, as its expression levels increased during the carcinogenesis period of PC [26]. Similarly, a significant correlation between *PLEC* expression levels and its copy number was identified in this study (Fig. 4d). *PLEC* expression levels were significantly lower in the C1 subgroup than in the C2/3/4 subgroups (Fig. 4e), and the frequencies of amplification and single gain were higher in the C2/3/4 subgroups (Fig. 4f). The above results could contribute to understand the better prognosis of C1 subgroup. In addition, the immune infiltration analysis based on the presence of six types of immune cells was performed in the four subgroups. After comparisons between C1 and C2/3/4 subgroups, the immune scores of macrophages, CD4 + T cell, and CD8 + T cell were found to be significantly higher in the C1 group. No obvious differences of B cell, myeloid dendritic cell, and neutrophil were identified among the four subgroups (Fig. 3c, d). These results indicated that the C1 group might be in an immunological enhanced state that could explain its better prognosis of PC.

### Results of GO and KEGG analyses based on genes corresponding to the CpG sites

**Fig. 5:**
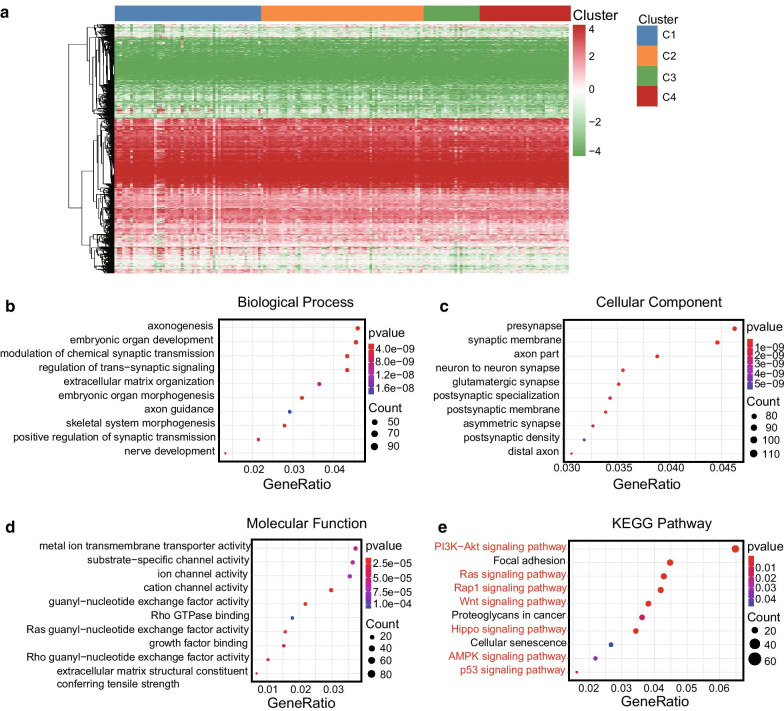
GO and KEGG analyses of 2939 genes corresponding to the 4227 CpG sites. **a** Heat map of the 2939 annotated genes of the total samples. **b** Top 10 pathways of the biological process enrichment analysis. **c** Top 10 pathways of the cellular component enrichment analysis. **d** Top 10 pathways of the molecular function enrichment analysis. **e** Top 10 pathways of the KEGG pathway enrichment analysis

To further investigate the underlying molecular mechanisms behind the prognosis-related subgroup division, we performed the GO and KEGG analyses based on the genes from the 4227 CpG sites, which were used for the consensus clustering. Firstly, gene annotations of the 4227 CpG sites were performed using the GRCh38 annotation file from the GENCODE project (https://www.gencodegenes.org). A total of 2939 genes were identified for further analysis, the heat map constructed based on the expression data of these genes is shown in Fig. 5a, and also detailed information is provided in Additional file 1: Table S5. The patterns of genes expression were different between the four subgroups, indicating that internal heterogeneity exists among them. The GO and KEGG analyses might contribute to better understand the molecular mechanisms behind the subgroups divisions. The biological process analysis identified several signal-related pathways including modulation of chemical synaptic transmission, regulation of trans-synaptic signalling, and positive regulation of synaptic transmission (Fig. 5b). The cellular component analysis showed several signalling pathways that could regulate the initiation and function of synapse (Fig. 5c), and several ion channel-related pathways were identified by the molecular function analysis (Fig. 5d). These results indicated that the signal transductions were significantly associated with the differences observed between the molecular subgroups. In addition, the results of KEGG analysis identified various classical tumour-associated pathways, such as PI3K-Akt, Ras, Rap1, Wnt, Hippo, AMPK, and P53 signalling pathway (Fig. 5e). The underlying molecular mechanisms behind the molecular characteristics of the subgroups will need to be further investigated in the future.

### Construction of a prognostic prediction model based on five CpG sites

**Fig. 6:**
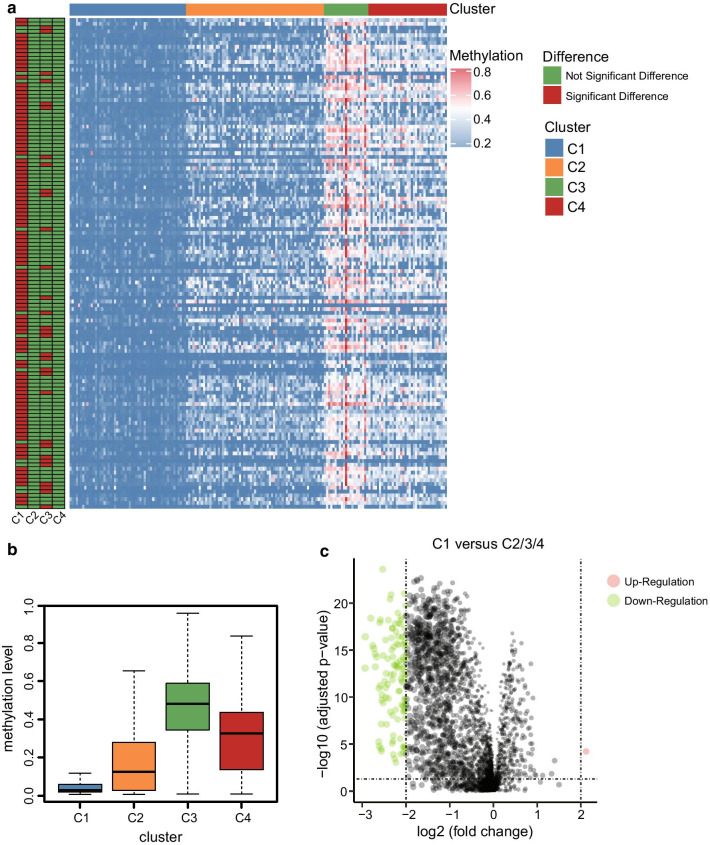
Differently methylated CpG sites among the four subgroups. **a** Heat map of the significant differently methylated CpG sites among the four subgroups. **b** Relationship between the methylation level and the different clustering subgroups. **c** Volcano plot of the differently methylated CpG sites between C1 and C2/3/4 subgroups

**Fig. 7:**
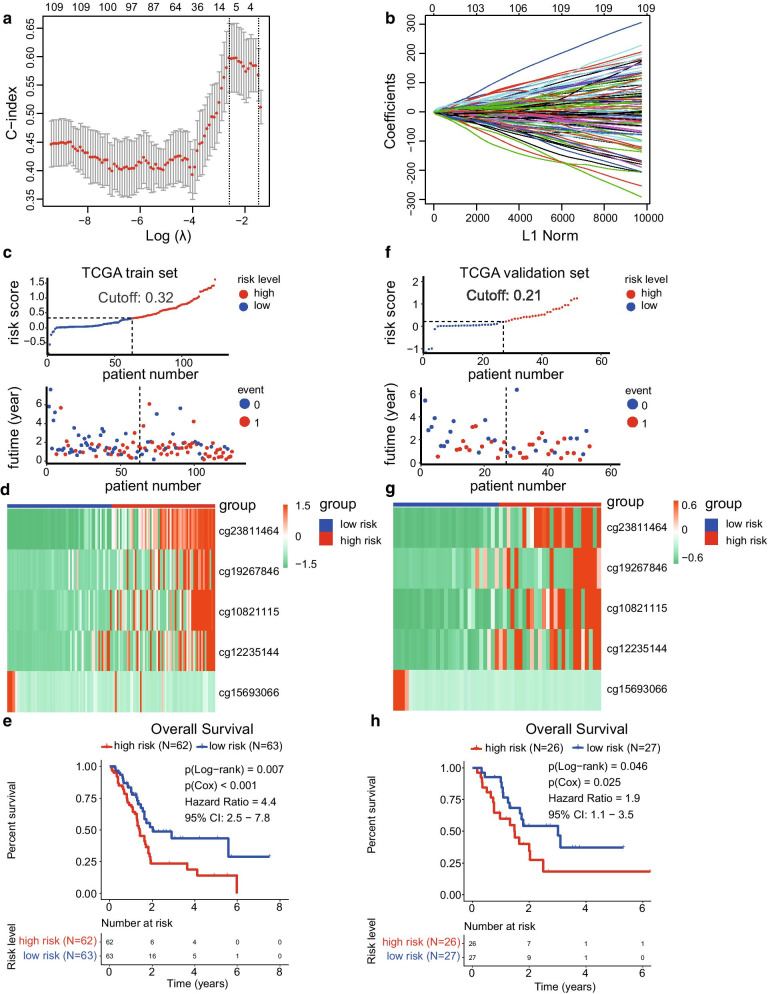
Development of a five-CpG-sites-based prognostic prediction model of pancreatic cancer using Lasso regression. **a** The λ value of the lasso model was decided by cross-validation with minimal misclassification. **b** Five CpG sites and their corresponding coefficients were utilised to construct the prognostic risk model. **c** Distribution plot of the risk score and survival time between high- and low-risk groups in the training set. **d** Heat map of the five CpG sites in the training set. **e** Overall survival analysis between the high- and low-risk groups in the training set. **f** Distribution plot of the risk score and survival time between high- and low-risk groups in the validation set. **g** Heat map of the five CpG sites in the validation set. **h** Overall survival analysis between the high- and low-risk groups in the validation set

To develop a specific tool for predicting the prognosis of PC patients, we decided to build a risk model based on the expression data of CpG sites. First, we calculated the differently methylated CpG sites between C1 and C2/3/4 subgroups, since the C1 group presented the best prognosis. After that, a total of 111 differently methylated CpG sites were obtained (|log2FC|> 2 and adjusted *P* value < 0.05). The volcano plot of the CpG sites is shown in Fig. 6c (Additional file 1: Table S6), and the heat map of the differently methylated CpG sites between each subgroup is shown in Fig. 6a. In addition, the boxplot of methylation levels of the four subgroups showed that C1 methylation level was relatively lower than the rest of the groups, while C3 group showed the highest methylation levels (Fig. 6b). The observed phenomena might contribute to the different prognosis of the molecular subgroups. To construct the prognostic prediction model, the total number of samples was randomly divided into training set (*N* = 125) and validation set (*N* = 53), and the Lasso model was used to construct the prognostic prediction model, which included cg23811464, cg19267846, cg10821115, cg12235144, and cg15693066, based on the 111 CpG expression sites data of the training set samples (Fig. 7a, b). The detailed information of the five CpG sites is listed in Table 1, and the risk score formula was depicted as follows:

- Risk score = cg23811464 × 1.40 + cg19267846 × 0.49 + cg10821115 × 0.76 + cg12235144 × 0.64 + cg15693066 ×  − 1.67

**Table 1:** Detailed information of the five CpG sites used to construct the risk score model

CpG sites	HR	Lower 95%CI	Upper 95%CI	*P* value	Coefficient
cg23811464	8.60	3.06	24.14	4.43E−05	1.40
cg19267846	6.79	1.55	29.70	0.011	0.49
cg10821115	5.76	2.05	16.17	0.001	0.76
cg12235144	4.27	1.69	10.82	0.002	0.64
cg15693066	3.68E−04	1.44E−07	0.94	0.048	− 1.67

**Fig. 8:**
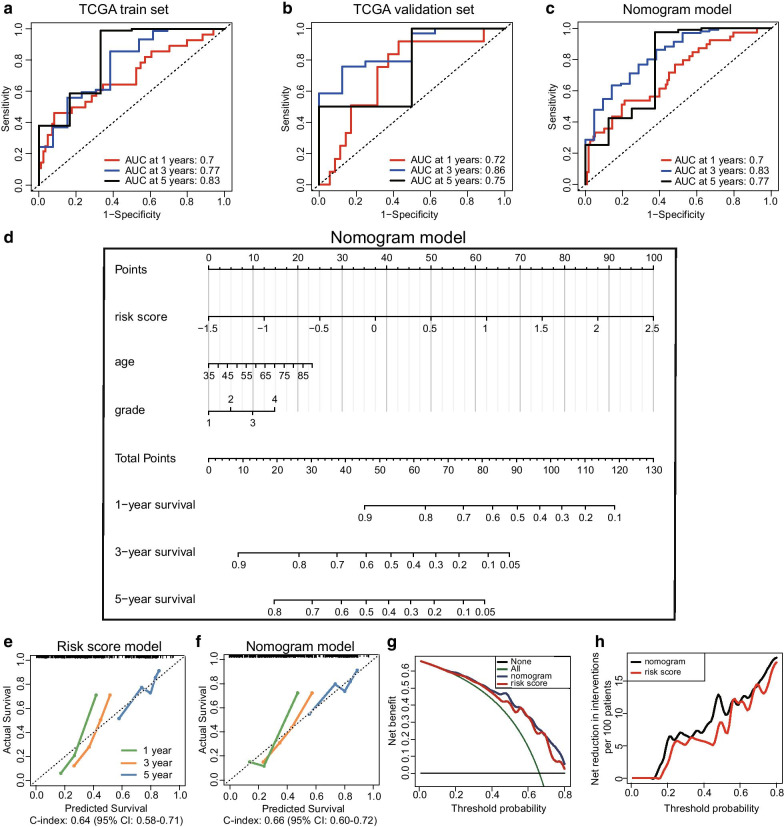
Development and evaluation of a nomogram model based on three independent prognostic factors. **a** ROC curve used to evaluate the 1-, 3-, and 5-year predictive efficiency of the five CpG sites model in the training set. **b** ROC curve used to evaluate the 1-, 3-, and 5-year predictive efficiency of the five CpG sites model in the validation set. **c** ROC curve used to evaluate the 1-, 3-, and 5-year predictive efficiency of the nomogram model. **d** Nomogram model constructed by using three independent prognostic factors including risk score model, age, and tumour grade. **e** Calibration curve of the risk score model. **f** Calibration curve of the nomogram model. **g**, **h** Decision curve analysis between the risk score and nomogram model. **g** The net benefit of the risk score model was lower than that of the nomogram model. **h** The net reduction in interventions per 100 patients of the nomogram model was higher than that of the risk score model

Each patient of the training set obtained a risk score by applying these formulas. The distribution diagram of the risk score and survival time among high-risk and low-risk groups is shown in Fig. 7c. Besides, the training set was separated into high-risk (*N* = 62) and low-risk groups (*N* = 63) by the median value of the risk scores. The heat map of the five CpG sites is shown in Fig. 7d. The overall survival analysis revealed significant difference between the high- and low-risk groups in the training set (Log − rank *P* value = 0.007, Cox *P* value < 0.001, HR = 4.4, 95% CI 2.5–7.8, Fig. 7e). Furthermore, the validation set was utilised to verify the validity and accuracy of the prognostic model based on the five CpG sites. Each sample of the validation set also acquired a risk score according to the same formula. The validation set was separated into high-risk (*N* = 26) and low-risk groups (*N* = 27) by the median value of the risk scores. The distribution plot of the risk score and survival time between high-risk and low-risk groups is provided in Fig. 7f. Besides, the heat map of the corresponding CpG sites is presented in Fig. 7g. Finally, the overall survival analysis between the high- and low-risk groups in the validation set also showed a significant difference (Log − rank *P* value = 0.046, Cox *P* value = 0.025, HR = 1.9, 95% CI 1.1–3.5, Fig. 7h). These results demonstrated that the prognostic prediction model based on the five CpG sites has good performance in both the train and validation set. The ROC curve was used to evaluate the efficiency of the five CpG signature sites. In the training set, the area under the curve (AUC) at years 1, 3, and 5 was 0.70, 0.77, and 0.83, respectively, while in the validation set was 0.72, 0.86, and 0.75, respectively (Fig. 8a, b). These results showed that the prognostic prediction signature based on the five CpG sites could be used as an efficient tool for predicting the prognosis of PC patients.

### Development of a novel nomogram model based on the independent prognostic factors

It is widely accepted that the nomogram model could be used as a reliable tool for the clinicians to make clinical decisions. In this study, the risk score model and other clinicopathological information were integratively utilised to construct a more effective and intuitive nomogram model. Firstly, the univariate Cox analyses of these factors demonstrated that the risk score model (*P* < 0.001), age (*P* = 0.008), grade (*P* = 0.006), T stage (*P* = 0.035), and N stage (*P* = 0.003) could serve as prognostic factors. Then, the multivariate Cox analyses identified that the risk score model (HR 8.114, 95% CI 3.674–17.918, *P* < 0.001), age (HR 1.032, 95% CI 1.004–1.061, *P* = 0.023), and tumour grade (HR 2.199, 95% CI 1.354–3.571, *P* = 0.001) were independent prognosis-related factors. The detailed results of the univariate and multivariate Cox analyses are provided in Table 2. Based on the data of the risk score model, age, and tumour grade, we developed the nomogram model to predict the survival rate of the PC patients after 1, 3, and 5 years (Fig. 8d). The AUC at years 1, 3, and 5 in the nomogram model was 0.70, 0.83, and 0.77, respectively (Fig. 8c). The calibration curves of the risk score and nomogram model showed that the two models present satisfied coherence between the actual survival and predicted survival rates. However, the C-index of the risk score model (C-index: 0.64, 95% CI 0.58–0.71) was lower than that of the nomogram model (C-index: 0.66, 95% CI 0.60–0.72) (Fig. 8e, f). To further compare the predictive efficiency between the risk score and nomogram models, we performed a decision curve analysis, and the results demonstrated that the nomogram model achieved a better performance (Fig. 8g, h). To sum up, the nomogram model developed based on the three-independent prognosis-related factors could serve as an efficient tool to predict the survival of PC patients.

**Table 2:** Univariate and multivariate Cox regression analyses of the prognostic factors

Prognostic factors	Univariate Cox analysis				Multivariate Cox analysis			
	HR	Lower 95%CI	Upper 95%CI	*P* value	HR	Lower 95%CI	Upper 95%CI	*P* value
Risk score	2.853	1.942	4.192	< 0.001	8.114	3.674	17.918	< 0.001
Age	1.027	1.007	1.048	0.008	1.032	1.004	1.061	0.023
Gender	0.832	0.559	1.238	0.364	0.701	0.359	1.368	0.298
Grade	1.472	1.116	1.943	0.006	2.199	1.354	3.571	0.001
Stage	1.328	0.940	1.875	0.107	0.999	0.213	4.685	0.999
T	1.573	1.034	2.395	0.035	0.695	0.168	2.877	0.616
M	1.108	0.341	3.602	0.865	1.427	0.056	36.071	0.829
N	2.128	1.285	3.527	0.003	2.140	0.860	5.325	0.102

## Discussion

PC is a highly lethal disease with a high level of genetic heterogeneity, and it has been shown that different histological subtypes exhibit distinct clinical, prognostic, and imaging outcomes [27, 28]. It is known that clinical treatment decisions are largely based on cancer clinical stage and pathological grade which are traditional prognostic factors with low efficiency. Nowadays, there is still a lack of personalised risk-adaptive therapeutic strategies for PC to meet the clinical demands. The identification of molecular subtypes of this malignancy has the potential to improve the prognostic and classification of PC. Considering the significant heterogeneity in the biological characteristics of PC, the current classification pattern relying solely on the classical clinicopathological features needs to be urgently improved.

Growing evidences have demonstrated that epigenetics plays an important role in heterogeneity. Epigenetic changes influence gene expression without altering the DNA sequence, and among them, the DNA methylation is the most in-depth epigenetic modification [29]. A wide range of genes are regulated by DNA methylation in different types of cancer [30]. Overall hypomethylation may lead to chromosomal instability, and hypermethylation is often associated with inactivation of tumour suppressor genes. Aberrant DNA methylation alters physiological homeostasis leading to tumourigenesis [31]. Moreover, the process of DNA methylation is reversible and therefore could serve as a potential therapeutic biomarker [32]. Recently, several scientific research groups have tried to explore the molecular subtypes of PC based on transcriptional profiles. These molecular classifications provided novel insights into the initiation and progression of PC from another perspective, revealing the existence of internal heterogeneity and the complexity of the tumour microenvironment. Therefore, understanding tumour heterogeneity and achieving a proper stratification of patients with cancer is still a major impediment to develop effective cancer therapy and to understand late and acquired therapy resistance [33].

In this study, a total of 178 PC patients were clustered into four distinct molecular subgroups based on their DNA methylation data and significant survival differences were identified among these groups. In addition, the molecular characteristics analyses (mutational spectrum, immune infiltration, GO, and KEGG) performed among these groups revealed new insights on PC development. These molecular subtypes may complement the previous histological classification of PC. The newly established prognostic risk model based on the five CpG sites (cg23811464, cg19267846, cg10821115, cg12235144, and cg15693066) could serve as a useful prediction tool of the prognostic of PC patients. Previously, three hypomethylated genes have been used to construct a prognostic prediction model using LASSO regression and the AUC of years 1, 3, and 5 which was 0.62, 0.69, and 0.69, respectively [34]. Zhou S. et al. proposed a prognostic signature model for PC patients based on the expression data of ANLN and HIST1H1C genes analysed by multivariate Cox regression, and the AUC of the two-gene model for 1 year was 0.673 [35]. The AUC of our five CpG sites model for 1 year, 3 years, and 5 years was 0.70, 0.77, and 0.83, respectively. These results indicated that the prognostic model based on the five CpG sites shows better performance than the two models previously reported. Besides, the nomogram model based on risk score model, age, and tumour grade could serve as a more efficient model compared with the risk model alone.

However, there are still some limitations in our present study. The study aimed to investigate the possibility to construct a prognostic prediction model, but the sample size available was relatively small and the results or conclusions should be revised in further studies using a larger sample size. The molecular classification model provides novel insights into the initiation and progression of PC, but the associated molecular mechanisms should be verified in by future research using in vitro or in vivo experiments.

### Conclusion

In this study, we established novel prognosis-related molecular subgroups based on the DNA methylation signature. Molecular characteristics and clinical feature comparisons among the four distinct subgroups provide a unique perspective on the occurrence and development of PC. The specific five CpG sites prognostic prediction model and derived nomogram model are effective and intuitive tools to assess the prognosis of PC patients. The identification of molecular subgroups based on DNA methylation data is an innovative complement to the traditional clinicopathological classification of PC and may contribute to the development of precision medicine, therapeutic efficacy prediction, and clinical decision guidance.

## Methods

### Data download

The DNA methylation data of PC patients generated from Illumina Human Methylation 450 platform were downloaded from the UCSC Xena platform [36]. The RNA sequencing (HTSeq–FPKM type), somatic mutation (MuTect2 Annotation), and copy number variation (Masked Copy Number Segment type) data of PC patients were downloaded from the Genomic Data Commons Data Portal of TCGA dataset (https://portal.gdc.cancer.gov). The most recent clinicopathological and follow-up information was obtained from TCGA website on 10 October 2020 [37]. The detailed description of the samples from TCGA dataset is provided in Additional file 1: Table S1.

### Data pre-processing

First, more than 70% of the missing CpG sites were excluded. Then, the CpG sites located in the sex chromosomes were also removed and the K-nearest neighbour (KNN) algorithm was utilised to estimate the not available (NA) data. We selected the CpG sites in the promoter regions (from the upstream 2 kb to the downstream 500 bp) for further analysis. In the RNA sequencing data, gene expression data lacking over 50% of the total sample information were excluded. PC samples with a survival time of more than 30 days were selected for further analysis.

### Identification of independent prognosis-related CpG sites

To find prognosis-related molecular subgroups based on DNA methylation, prognosis-related CpG sites should be identified. Firstly, the CpG sites expression data and survival information were merged and the univariate Cox proportional hazards regression survival analysis was used to select the significantly prognostic CpG sites (*P* < 0.05, Additional file 1: Table S2). Then, the multivariate Cox proportional hazards regression model was used to select the independent prognostic CpG sites from the results of the univariate Cox analysis by combing the clinicopathological information available, including age, gender, tumour grade, clinical stage, T stage, M stage, and N stage (*P* < 0.05). A total of 4227 independent prognostic CpG sites were identified for further studies. Detailed information is provided in Additional file 1: Table S3.

### Identification of prognosis-related molecular subgroups

To establish the prognosis-related molecular subgroups, we performed the consensus clustering analysis from the R package ‘Consensus Cluster Plus’, according to the official guide [38]. The number of clusters of the total PC samples was defined by the Consensus Cumulative Distribution Function (CDF) Plot. When the K value was set to 4, the area under the CDF almost reaches its maximum value. Therefore, the total samples were divided into four groups.

### Survival analysis between subgroups

The progression-free interval (PFI) and overall survival (OS) analyses were performed between the subgroups, and the results were visualised using the R package ‘survival’ and ‘survminer’ [39–41].

### Molecular characteristics analyses between subgroups

To analyse the molecular characteristics of the four identified subgroups, we performed the mutation spectrum analysis using the R package ‘maftools’, according to the official guideline [42]. The TIMER2.0 website (http://timer.cistrome.org) was used to analyse the presence of six types of tumour-infiltrating immune cells between the different subgroups [43–45]. The variations on the copy numbers, including amplification and deletion of specific genes, between the subgroups were analysed. The R packages ‘clusterProfiler’, ‘enrichplot’, and ‘ggplot2′ were used to perform the Gene Ontology (GO) and KEGG (Kyoto Encyclopedia of Genes and Genomes) pathways analyses [46–48].

### Construction and evaluation of a prognostic risk model based on five CpG sites

The Wilcox test was used to calculate CpG sites differential expression between the best prognosis subgroups, C2/3/4 and C1. The criteria were defined as |log2FC|> 2 and adjusted *P* value < 0.05, and a total of 111 CpG sites were obtained. (Detailed information is provided in Additional file 1: Table S6.) The total sample was randomly divided into 70% (N = 125) and 30% (N = 53), which were used as the training set and validation set, respectively. Then, the least absolute shrinkage and selection operator (Lasso) method was used to construct the prognostic risk model based on five CpG sites (cg23811464, cg19267846, cg10821115, cg12235144, and cg15693066) and the training set. The Lasso method was performed with the R package ‘glmnet’ [49, 50]. The validation set was used to verify the reliability and suitability of the prognostic risk model. The receiver operating characteristic (ROC) curve was used to evaluate the predictive efficiency of the prognostic risk model at years 1, 3, and 5, using the R package ‘timeROC’ [51].

### Establishment of the nomogram model based on the independent prognostic factors

To improve the prognostic predictive efficiency of the model in PC patients, the univariate and multivariate Cox analysis was performed to identify independent prognostic factors based on the risk model previously described and clinicopathological data. Risk model, age, and tumour grade were the three factors used to build the nomogram model with the R packages ‘rms’ and ‘regplot’ [52, 53]. The discriminative efficiency was assessed by calibration curve and decision curve analyses [54, 55].

### Statistical analysis

The R software version 3.6.1 and RStudio software were used to perform the statistical analyses and figures output.

## Supplementary Information

**Additional file 1.** **Table S1:** The detailed description of the samples from TCGA dataset. **Table S2:** Detailed information of 29,879 prognosis-related CpG sites. **Table S3:** Detailed information of 4,227 independent prognostic CpG sites. **Table S4:** Detailed information of genes with different mutational frequency between the C1 and C2/3/4 subgroups. **Table S5:** The expression data of 2,939 genes annotated from the 4,227 independent prognostic CpG sites. **Table S6:** Detailed information of the 111 differently methylated CpG sites between the C1 and C2/3/4 subgroups.

## Abbreviations

TCGA: The Cancer Genome Atlas
KNN: K-nearest neighbour
CDF: Consensus cumulative distribution function
PFI: Progression-free interval
OS: Overall survival
GO: Gene ontology
KEGG: Kyoto Encyclopedia of Genes and Genomes
ROC: Receiver operating characteristic
